# A173 EVALUATING VACCINATION IN INFLAMMATORY BOWEL DISEASE – A QUALITY ASSESSMENT STUDY IN THE WINNIPEG HEALTH REGION

**DOI:** 10.1093/jcag/gwab049.172

**Published:** 2022-02-21

**Authors:** P Kundapur, A Ilnyckyj

**Affiliations:** University of Manitoba, Winnipeg, MB, Canada

## Abstract

**Background:**

The Canadian Association of Gastroenterology published a two-part clinical practice guideline, highlighting that IBD patients may be more susceptible to vaccine preventable diseases. Various studies have demonstrated shortcomings in vaccination rates amongst IBD patients. It is imperative to ensure that an individual’s vaccination status be known prior to starting immunosuppressive therapy; and deficiencies be addressed to optimize patient outcomes.

**Aims:**

Identify physician perceived barriers to vaccinations in the IBD cohort.

Determine discrepancies in vaccination rates in IBD patients, pre and post-introduction of the clinical tool.

**Methods:**

Gastroenterologists in the Winnipeg Health Region (WRHA) will be provided with a survey to assess barriers to vaccination. This survey will be used to inform the clinical tool that will be developed to support physicians. The study is divided into a retrospective component (“pre-clinical tool”) and a prospective component (“post-clinical tool”). In the pre-clinical tool assessment, patient charts of new biological starts at St. Boniface Hospital (SBGH); between January – December 2020, will be reviewed to determine vaccination discussions. For the post-clinical tool assessment, SBGH Gastroenterologists will be provided a document containing guideline recommended vaccination schedule for IBD patients. Subsequently, vaccination rates in IBD patients starting biological therapy will be evaluated over the following 3 months.

**Results:**

Response rates were 90% for the 10 WRHA Gastroenterologists surveyed. Only 55% felt comfortable providing vaccine related care to their IBD patients. Despite being comfortable, 40% correctly identified recommended annual vaccination and 60% accurately identified vaccines that were contraindicated in IBD patients on immunosuppressive therapy. 55% would assess vaccination status with their IBD patients “sometimes,” and 33% state they have “never” provided age-appropriate vaccinations prior to initiation of immunosuppression. Physician related factors (Physician Knowledge) was the most commonly cited obstacle faced in providing immunization care. 80% believed that Primary Care Physicians should keep track and be responsible for administering vaccines to IBD patients. 88% specified that having a clinical tool describing vaccine recommendations would be useful in their practice. Retrospective chart review is in progress and prospective data collection is scheduled to be completed in February 2022.

**Conclusions:**

Survey analysis demonstrates that vaccine practices in WRHA are inadequately addressed; the leading obstacle for physician related factors was knowledge. Though chart review is underway, we anticipate that current vaccination assessments in IBD patients undergoing biological starts are low. We anticipate that rates of vaccinations will improve with the provision of a clinical tool.

**Funding Agencies:**

None

